# Evaluation of potential solid organ donors in São Paulo, Brazil: Reasons for non-donation and the impact of infectious causes

**DOI:** 10.1016/j.clinsp.2026.101171

**Published:** 2026-09-30

**Authors:** Luiza Arcas Gonçalves, Edvaldo Leal de Moraes, Maristela Pinheiro Freire, Leonardo Borges de Barros e Silva, Wellington Andraus, Luiz Augusto Carneiro D' Albuquerque, Edson Abdala, Alice Tung Wan Song

**Affiliations:** a Departamento de Infectologia e Medicina Tropical, Faculdade de Medicina, Universidade de São Paulo, São Paulo, SP, Brazil; b Organização de Procura de Órgãos do Hospital das Clínicas da Faculdade de Medicina da Universidade de São Paulo, São Paulo, SP, Brazil; c Comissão de Controle de Infecção Hospitalar, Hospital das Clínicas, Faculdade de Medicina, Universidade de São Paulo, São Paulo, SP, Brazil; d Disciplina de Transplante de Fígado e Órgãos do Aparelho Digestivo, Departamento de Gastroenterologia, Hospital das Clínicas, Faculdade de Medicina, Universidade de São Paulo, São Paulo, SP, Brazil

**Keywords:** Solid organ donors, Infectious disease, Donor evaluation, Non-donation, Organ procurement

## Abstract

**Background:**

Solid organ transplantation is a life-saving therapy for patients with end-stage organ disease; however, the persistent imbalance between organ demand and supply substantially increases waiting-list mortality. Expanding the donor pool is essential but must be balanced with safety and minimization of infectious risk. This study aimed to describe the main reasons for organ non-donation, with a particular focus on infectious causes, and to identify factors associated with procurement failure.

**Methods:**

We conducted a retrospective cohort study using secondary data from the Organ Procurement Organization at Hospital das Clinicas, University of São Paulo, from 2019 to 2023. Monthly rates of donor exclusion due to infectious causes were evaluated, with comparisons between pandemic and non-pandemic periods. Factors associated with non-donation due to infection were assessed using Poisson regression with robust variance. The COVID-19 pandemic period was defined as March 2020 to March 2022.

**Results:**

A total of 2591 potential solid organ donors were evaluated. Medical contraindications were the leading cause of non-donation, predominantly driven by infectious causes. The proportion of infection-related non-donation increased significantly during the pandemic compared with the non-pandemic period (45.3% vs. 28.2%; *p* < 0.001). White ethnicity, intensive care unit admission, and trauma or vascular causes of death were identified as protective factors against non-donation due to infection. The most frequent infectious causes were COVID-19, uncontrolled bacterial infections, HIV and tuberculosis.

**Conclusions:**

Infectious diseases represent a potentially modifiable cause of non-donation. These findings highlight the need for continuous reassessment of donation-related infectious risks and evidence-based donor infection management to prevent unnecessary organ loss.

## Highlights

• Fewer than one third of potential donors become actual donors.
• Medical contraindications are the main cause of non-donation.
• Infections are the leading cause of organ donation exclusion.
• The pandemic increased non donation due to infections.
• Infection risk remains a key barrier to transplantation.

## Introduction

Solid Organ Transplantation (SOT) is a therapeutic option for patients with advanced organ diseases. Since 1997, Brazil has implemented a centralized system for organ procurement and distribution, enabling nationwide access to transplantation through the Brazilian Unified Health System (SUS).^1^^,^^2^ The Brazilian National Transplant System is coordinated by the Ministry of Health with national governance but implemented through a decentralized regional structure composed of State Transplant Centers. The transplant system is universal and free of charge, operating under a single national waiting list managed by an electronic information system that allocates organs according to technical and logistical criteria of compatibility and urgency. State centers are responsible for local organ allocation, and when an organ is not used locally, it is offered to the National Transplant Center for interstate redistribution.^3^ This system has established Brazil as a global leader in transplantation, ranking as the fourth-highest country in the number of liver and kidney transplants performed in 2023. Despite its high absolute number of transplants, Brazil's transplant rates per million population (pmp) remain relatively low, with 28.3 kidney transplants and 10.9 liver transplants pmp.^4^

In 2024, there were 36,985 active patients on the kidney transplant waiting list in Brazil. A total of 6,297 transplants were performed during the year, representing approximately 17% of those on the waiting list, while 2,606 patients died while waiting for an organ.^5^ In Brazil, organ donation from deceased donors predominates, however, this supply remains insufficient. In 2024, 15,090 potential donors were evaluated, but only 4,088 became effective donors, further widening the gap between organ demand and availability.^4^ Among the main causes for non-donation in Brazil, family refusal is the most frequent, a particularly relevant issue given that organ donation after death legally depends on family authorization.

The second most frequent cause of non-donation is medical contraindication, which occurs when the risk of disease transmission outweighs the potential benefits for the recipient, particularly in cases involving neoplasms or infectious conditions. In Brazil, absolute contraindications for organ donation include HIV seropositivity, HTLV I and II seropositivity, active tuberculosis, certain neoplasms (except primary central nervous system tumors and in situ carcinomas of the uterus or skin), refractory sepsis, and severe or potentially severe viral infections in the presence of immunosuppression (e.g., rubella, rabies, West Nile virus, adenovirus, enterovirus, parvovirus, and viral meningoencephalitis of unknown origin). Other conditions, such as cryptococcal meningoencephalitis and prion diseases, also preclude donation.^6^^,^^7^

To ensure the safety of SOT recipients, a thorough evaluation of potential donors is essential to mitigate the risk of disease transmission. This process involves a comprehensive assessment of the donor's medical history, clinical examination, and serological screening tests. In Brazil, this includes testing for HIV, HTLV I and II, HBsAg, Anti-HBs, total Anti-HBc, Anti-HCV, syphilis, and Chagas.^7^ Assessing the infectious profile of potential donors plays a key role in devising strategies to increase organ recovery, particularly in contexts where the disparity between organ demand and supply significantly increases the mortality risk for patients awaiting transplantation. The objective of this study was to describe the main reasons for organ non-donation in a region of the State of São Paulo, Brazil, with a particular focus on infectious causes, and to identify factors associated with procurement failure.

## Materials and methods

### Study design

This retrospective cohort was conducted with secondary data from the Organ Procurement Organization (OPO) at the Hospital das Clinicas, Faculdade de Medicina, University of São Paulo. The OPO, a supra-hospital organization, coordinates organ and tissue donation processes, maintains potential donors, and provides training to support the identification and realization of donations in accordance with the National Transplant System's regulations. Responsible for the western zone of São Paulo city, with a population of 7,544,088, this OPO accounted for 201 of the 1,023 effective organ donors in the state in 2024 – nearly 5% of Brazil’s total of 4,088 donors. The data was obtained by the OPO from donor information forms and records, which are routinely collected as part of the organ procurement process. The analysis covered a 5-year period from 2019 to 2023.

The donor information form is a standardized template provided by the São Paulo State Transplant System. It includes detailed demographic and medical history of the potential donor, a summarized account of the brain death process, and the final cause of death. Additional information was provided by the OPO regarding the donation evaluation process, including clinical assessment, family consent, and the subsequent execution of the transplant, detailing the utilized organs.

Collected variables included sex at birth, age, ethnicity/color, and the cause of brain death, with a particular focus on infectious causes. The percentage of central nervous system infections leading to brain death was detailed, encompassing bacterial, viral, mycobacterial, parasitic, and unknown etiologies. Reasons for donation non-proceeding were analyzed and classified into four categories: family refusal, cardiorespiratory arrest, medical contraindications, and other reasons. The proportion of each cause for non-donation was obtained by dividing the number of cases in each category by the total number of potential donors. Medical contraindications were further analyzed to evaluate the contribution of infectious causes. Additionally, reasons for organ discards were assessed. Organ discard was defined as the recovery of an organ from a donor that was subsequently not transplanted into a recipient. For analyses involving the COVID-19 pandemic, the period was defined as March 2020 to March 2022, when the expanded use of COVID-19–positive donors began in Brazil.^8^

### Data analysis

Categorical variables were described as absolute numbers and percentages, while numerical variables were summarized using medians and interquartile ranges. A polynomial trend curve was used to analyze temporal trends. The main outcome was the monthly rate of donor exclusion due to infectious causes, defined as the number of potential donors medically contraindicated for organ donation due to infection, divided by the total number of potential donors evaluated each month. A descriptive analysis of infection-related causes of non-donation was also performed, comparing the pandemic period (March 2020 to March 2022) with the non-pandemic period (2019 to February 2020 and April 2022 to 2023). A chi-square test was applied to compare the frequency of infection-related non-donation between these periods. Bivariate and multivariate analyses of risk factors for non-donation due to infectious causes were conducted using Poisson regression with robust variance. Data analysis was performed using STATA version 17. This observational study was conducted and reported in accordance with the Strengthening the Reporting of Observational Studies in Epidemiology (STROBE) Statement. The study was approved by the Research Ethics Committee of the Hospital das Clínicas, University of São Paulo School of Medicine, on April 4, 2024 (CAAE: 79022124.6.0000.0068).

## Results

A total of 2,591 potential organ donors were evaluated during the study period. The annual distribution was as follows: 464 (17.9%) in 2019, 443 (17.1%) in 2020, 487 (18.8%) in 2021, 621 (23.9%) in 2022, and 576 (22.2%) in 2023. Most evaluations occurred in the Intensive Care Unit (ICU), accounting for 2,011 cases (77.6%), followed by the emergency department with 559 cases (21.5%), and the semi-intensive care unit with 17 cases (0.6%). The median age of potential donors was 50-years, with an interquartile range of 36 to 62 years. Most of the potential donors were men (56.2%), with more than half identifying as non-white (54.7%), as presented in the sex and ethnicity data in Fig. 1.

**Fig. 1:**
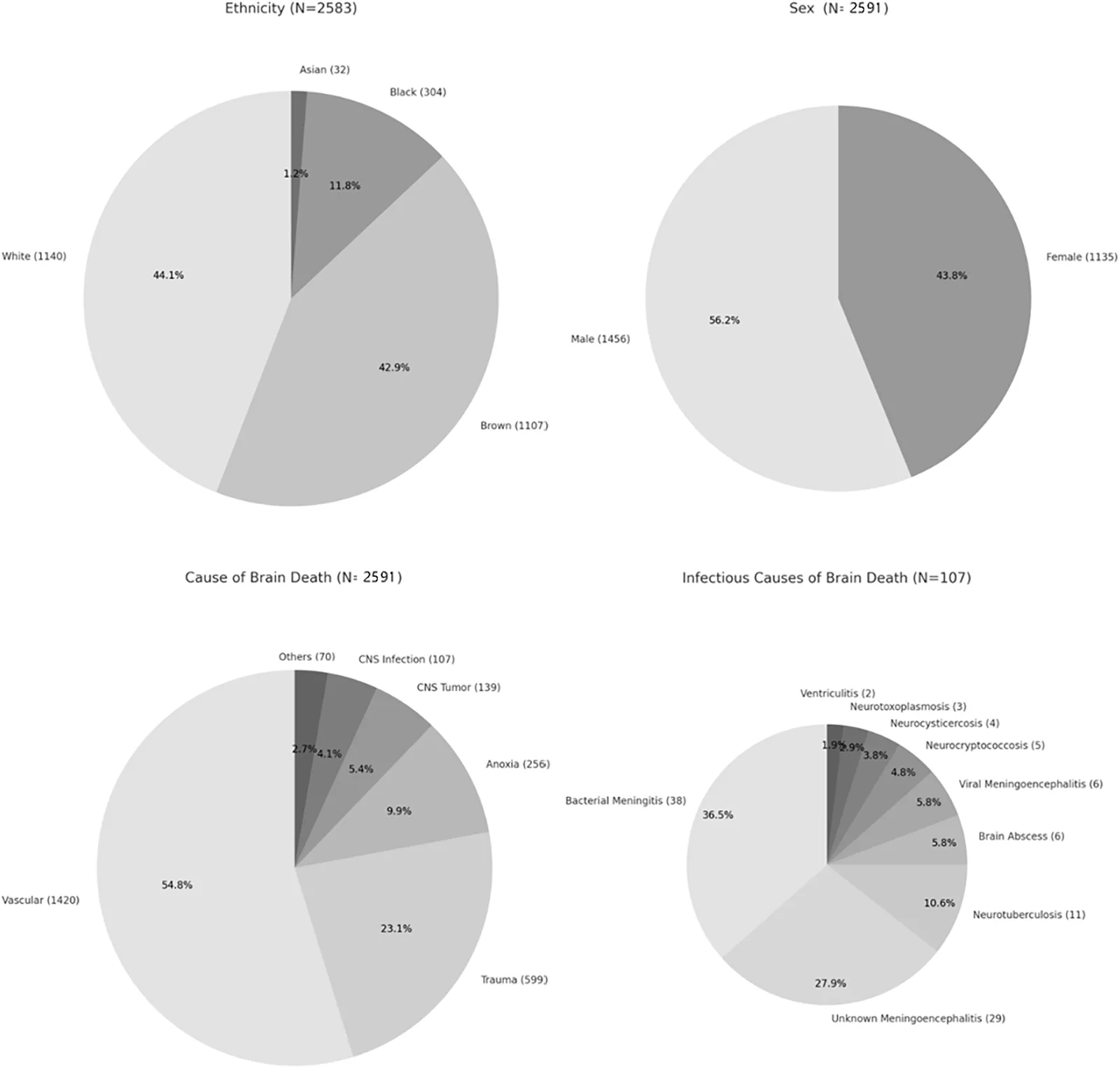
Distribution of 2591 potential organ donors according to sex, ethnicity, causes of brain death, and types of infections leading to brain death.

The causes of brain death were predominantly vascular events (54.8%), followed by trauma (23.1%), anoxia (9.8%), Central Nervous System (CNS) tumors (5.4%), CNS infections (4.1%), and other causes (2.7%). Among the cases of brain death caused by infections, bacterial meningitis and meningoencephalitis of unknown origin were the most frequent causes, accounting for 38 (36.5%) and 29 (27.9%) of cases, respectively. Other infectious causes included neurotuberculosis, cerebral abscess, viral meningoencephalitis, neurocryptococcosis, neurocysticercosis, neurotoxoplasmosis, and ventriculitis. Detailed data are presented in Fig. 1.

Of the 2,591 potential donors evaluated, only 27.5% (*n* = 713) had at least one organ transplanted, while 1,878 did not proceed to donation. The main reason for procurement failure was medical contraindications (*n* = 860), followed by family refusal (*n* = 572). Among the 860 cases with medical contraindications, specific reasons were identified for 600 cases. For the remaining cases, no justification was reported in the donor information forms or in the official records of the OPO. Infections were the leading cause of contraindication, accounting for 305 cases (35.4%), followed by neoplasms (149 cases, 17.3%). Among the 305 cases contraindicated due to infections, the most frequent causes included COVID-19 (96 cases, 31.5%), uncontrolled bacterial infections (51 cases, 16.7%), HIV (44 cases, 14.4%), and meningoencephalitis of unknown origin (31 cases, 10.2%). Regarding organ discards, the main cause was organ morphological alterations (80 cases, 70.1%), followed by age (12 cases, 10.5%). The data are detailed in Table 1, Table 2.

**Table 1:** Outcomes and reasons for non-donation, including medical contraindications and discard causes.

Outcome	*n* = 2591
**Actual organ donation**	**713 (27,5%)**
**Non-Donation**	**1878 (72,5%)**
**Medical contraindication**	**860 (33.2%)**
Infection	305 (35.4%)
Neoplasia	149 (17.3%)
Severe organ dysfunction	58 (6.7%)
Uncertain cause of death	43 (5.0%)
Age	42 (4.8%)
Comorbidity	13 (1.5%)
Unknown	260 (30.2%)
**Family refusal**	**572 (22.0%)**
**Cardiorespiratory arrest**	**283 (10.9%)**
**Discard**	**114 (4.4%)**
Morphological alteration	80 (70.1%)
Age	12 (10.5%)
Comorbidity	11 (9.6%)
Neoplasia	6 (5.2%)
Cardiorespiratory arrest	6 (5.2%)
**Others**	**49 (1.9%)**

**Table 2:** Infectious causes of medical contraindications to organ donation.

Contraindications Due to Infection	2019‒2023	Pandemic period	Non-pandemic period
	*n* = 305	*n* = 165	*n* = 140
COVID-19	96 (31.5%)	91 (55.2%)	5 (3.6%)
Uncontrolled bacterial infection	51 (16.7%)	23 (13.9%)	28 (20.0%)
HIV	44 (14.4%)	18 (10.9%)	26 (18.6%)
Unknown meningoencephalitis	31 (10.2%)	2 (1.2%)	29 (20.7%)
Tuberculosis	25 (8.2%)	6 (3.6%)	19 (13.6%)
Hepatitis C	14 (4.6%)	8 (4.8%)	6 (4.3%)
Chagas disease	11 (3.6%)	4 (2.4%)	7 (5.0%)
Multidrug-resistant infection	10 (3.3%)	5 (3.0%)	5 (3.6%)
Severe viral infection	6 (2.0%)	0	6 (4.3%)
Fungal meningoencephalitis	6 (2.0%)	2 (1.2%)	4 (2.9%)
HTLV	6 (2.0%)	3 (1.8%)	3 (2.1%)
Hepatitis B	3 (1.0%)	0	3 (2.1%)
Others	5 (1.6%)	1 (0.6%)	4 (2.9%)

COVID-19, Coronavirus Disease 2019; HIV, Human Immunodeficiency Virus; HTLV, Human T-lymphotropic Virus.

During the non-pandemic period, 1,596 potential donors were evaluated, of whom 445 became effective donors with organs transplanted. There were 496 medical contraindications, 140 of which were due to infectious causes (28.2%). In contrast, during the pandemic period, 995 potential donors were evaluated, of whom 268 had organs transplanted. A total of 364 donors were medically contraindicated, including 165 due to infectious causes (45.3%). A Chi-Square test showed a significant association between the pandemic period and non-donation due to infection (45.3% vs. 28.2%; χ^2^ = 26.09; *p* < 0.001), indicating an increase during the pandemic. The causes of non-donation due to infection are detailed for each period in Table 2 and the graphical comparison between periods is illustrated in Fig. 2.

**Fig. 2:**
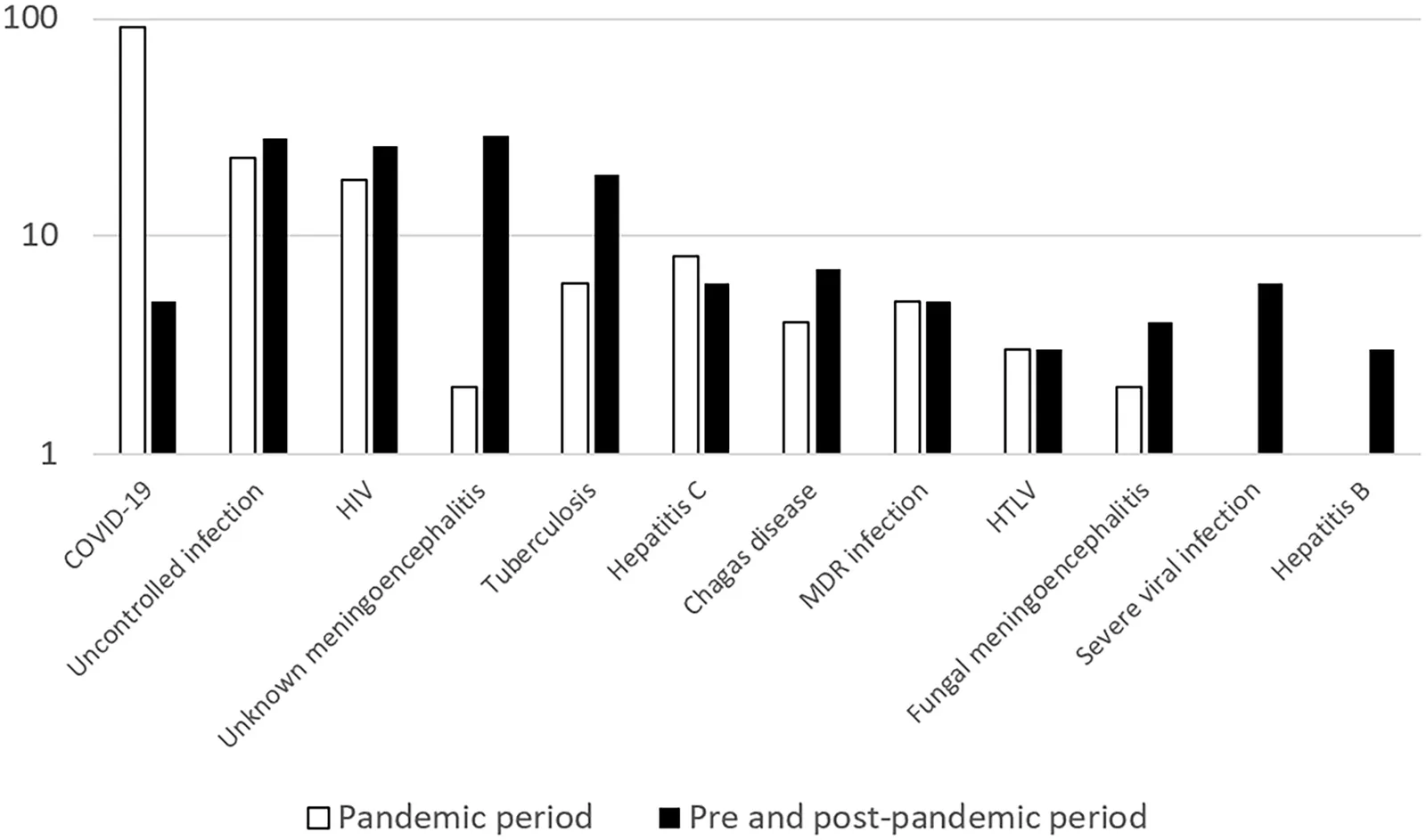
Comparison of infectious causes of medical contraindications between pandemic and pre- and post-pandemic periods.

The rate of donor exclusion due to infection was evaluated throughout the study period, showing an increasing trend from early 2020 to mid-2022 ‒ a timeframe consistent with the Coronavirus Disease 2019 (COVID-19) pandemic, as shown in Fig. 3. Factors associated with donor exclusion due to infection were assessed and are presented in Table 3. Considering the entire study period, white ethnicity, ICU admission, and trauma or vascular causes of death were identified as protective factors against non-donation due to infection, whereas older age and donor evaluation during the COVID-19 pandemic were associated with an increased risk of non-donation due to infection. Female sex was excluded from the final multivariable model because it was not significantly associated with the outcome (IRR = 0.922, 95% CI 0.786‒1.080, *p* = 0.318). In the analysis restricted to the non-pandemic period, white ethnicity was the only factor identified as protective against non-donation due to infection. In contrast, during the pandemic period, female sex was identified as a protective factor, whereas white ethnicity was associated with an increased risk of non-donation due to infection.

**Fig. 3:**
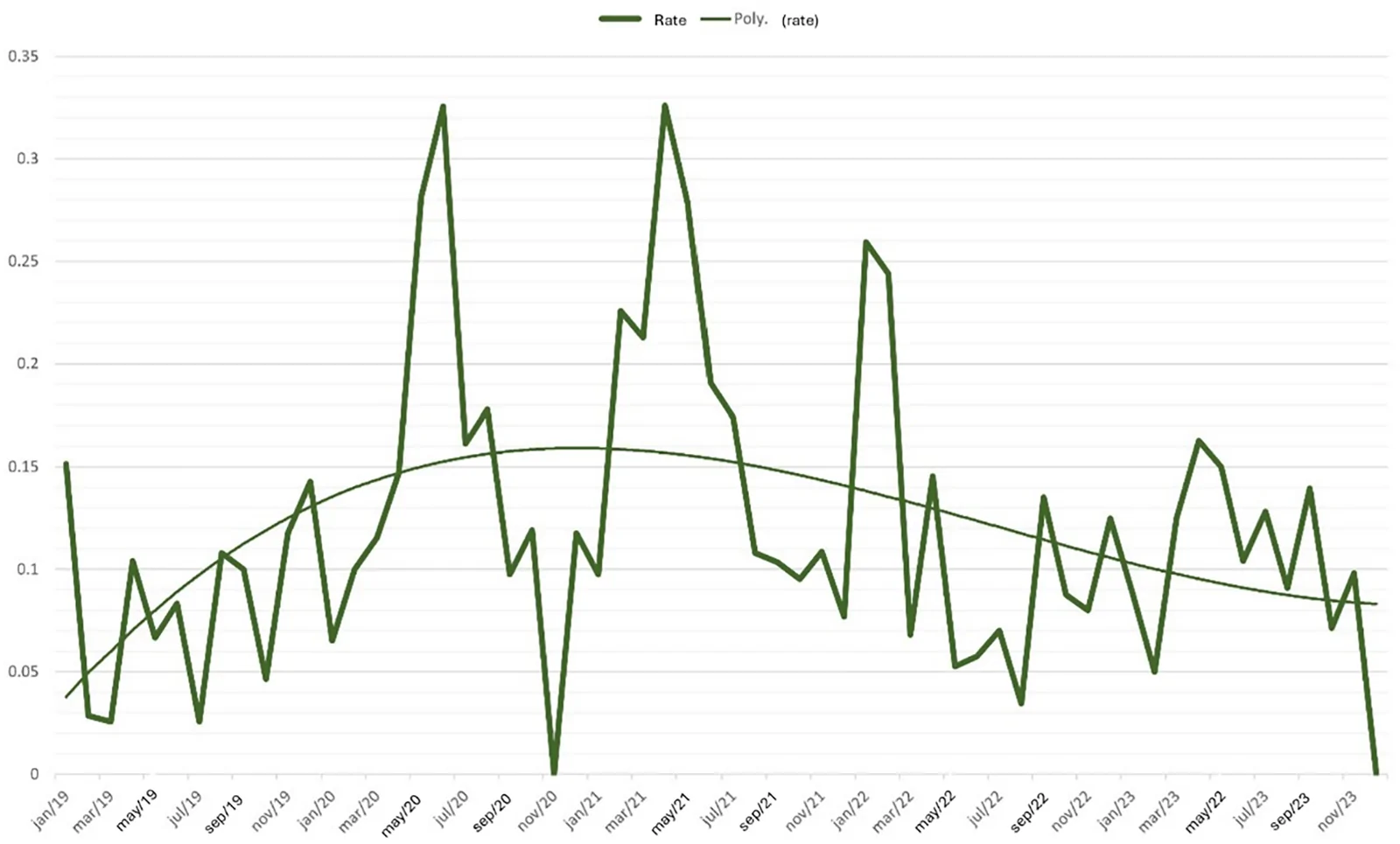
Temporal trends of the donor exclusion rate due to infectious causes.

**Table 3:** Multivariable analysis of risk factors for non-donation due to infection.

Variable	IRR	95% CI	*p*-value^a^
**Period 2019‒2023**			
**Evaluation at ICU Unit**	0.640	0.583–0.702	0.000
**White Ethnicity**	0.511	0.433–0.603	0.005
**Age in years**	1.277	1.118–1.459	0.000
**COVID-19 Pandemic**	4.900	1.261–19.032	0.022
**Cause of Brain Death**			
Trauma	0.511	0.433–0.603	0.000
Vascular	0.569	0.483–0.672	0.000
**Non-pandemic period**			
**White Ethnicity**	0.94	0.92–0.97	<0.001
**Pandemic period**			
**Female sex**	0.922	0.89–0.96	<0.001
**Trauma-related brain death**	0.953	0.91–1.00	0.05
**White Ethnicity**	1.05	1.01–1.09	0.01

IRR, Incidence Rate Ratio; 95% CI, 95% Confidence Interval; ICU, Intensive Care Unit; COVID-19, Coronavirus Disease 2019.

a Statistical significance based on Pearson's Chi-Square test or Fisher's exact test.

## Discussion

In this original retrospective study, we have shown that the rate of procurement failure in a region from Brazil is high, with the main causes being medical contraindications followed by family refusal. Infections emerged as the most prevalent cause of medical contraindication, being especially prominent during the COVID-19 pandemic but still representing an important cause in the pre-and post-pandemic periods. Evaluating the factors associated with the donor exclusion due to infection can help develop strategies to improve organ donation.

Expanding the donor pool is crucial for increasing organ availability and improving the survival of those on waiting lists. One approach is the adoption of more flexible donor selection criteria, which can directly impact the increase in effective donors. This includes the use of expanded criteria donors, such as older individuals and those with comorbidities, as well as the acceptance of nonstandard risk donors, such as those with a history of malignancy or active infection, despite their potentially higher transmission risk.^9^^,^^10^ A thorough evaluation of the potential donor is necessary to determine whether the assumed risk can be tolerated and if prophylactic and interventional measures can be implemented.^11^

For this purpose, in addition to assessing clinical and personal history, laboratory screening is performed, including testing for HIV, HTLV I and II, HBsAg, Anti-HBs, total Anti-HBc, Anti-HCV, syphilis, and Chagas disease.^7^ The assessment of the infectious risk is dynamic, evolving with the accumulation of scientific knowledge over years of transplantation experience, as well as advancements in screening, prophylaxis, and interventions to mitigate transmission risk. Notably, in our sample, infections were identified as the leading cause of medical contraindication for organ donation. Although some infections are indeed absolute contraindications at present, such as active tuberculosis or meningoencephalitis of unknown origin, others may be more carefully reconsidered, such as COVID-19, Chagas disease, hepatitis C, multidrug-resistant agents, and uncontrolled bacterial infections.

The use of hepatitis C viremic donors, for example, represents a change enabled by the approval and efficacy of Direct-Acting Antivirals (DAAs) and evidence demonstrating the safety of using viremic patients in solid organ transplantation without differences in survival, including liver transplants.^12^^,^^13^ In the state of São Paulo, where the present study was conducted, since March 2020,^14^ there has been authorization for the distribution of organs from hepatitis C viremic donors to be used regardless of the recipient’s virus C viremia, ensuring that the recipient undergoes appropriate treatment. Our findings indicate that donor exclusion due to hepatitis C accounted for 4.6% contraindications related to infectious diseases, occurring during a transitional period in the acceptance of hepatitis C viremic donors. This highlights the importance of continuously reassessing the risk of donor-transmitted infections and the acceptance of non-standard risk donors.

Likewise, COVID-19 emerged as the leading cause of non-donation due to infectious reasons. The impact of the COVID-19 pandemic on solid organ donation and transplantation activity was characterized by a significant decline, particularly during the second wave of the disease in early 2021.^15^ Consistently, our study found an increase in the donor exclusion rate during the pandemic. At the beginning of 2020, with the emergence of a new infection and the lack of knowledge about its impact on transplant outcomes, regulatory agencies and disease control and prevention authorities, such as the European Centre for Disease Prevention and Control and the Ministry of Health of Brazil, contraindicated the organ use of confirmed or suspected COVID-19 donors.^16^ Throughout the pandemic, studies involving transplant recipients from COVID-19-positive donors demonstrated no differences in graft survival or mortality when compared to COVID-19-negative donors.^17^, ^18^, ^19^ This evidence led to revised recommendations regarding the use of SARS-CoV-2 – positive deceased donor organs, with increased permissiveness except for lung and intestine transplants.^20^

Following SARS-CoV-2 infection, uncontrolled infections were identified as the second leading cause of contraindication for potential donors due to infectious diseases. While infection plays a minimal role as a direct cause of brain death, this study revealed that nearly half of all medical contraindications to organ donation were attributed to infectious diseases. Prior studies in Brazil had already identified infection as the second most common reason for non-donation, second only to family refusal,^21^ emphasizing the significant impact of infection-related donor exclusions. In situations where infection is not the primary cause of death, it is hypothesized that infectious complications during the management of critically ill patients may contribute to the increased frequency of donor exclusion due to uncontrolled infections. Preventing and effectively managing infections could help reduce these rates, as donors with ongoing infections may still be accepted if the infectious process is controlled with at least 48-hours of appropriate antimicrobial therapy, followed by extending therapy to the recipient for a minimum of seven days.^22^

Given the greater difficulty in treating infections caused by Multidrug-Resistant (MDR) bacteria, there is increased concern regarding the acceptance of potential donors colonized or infected with these agents. However, in the context of donor shortages, further restricting acceptance may negatively impact organ availability. Although donor infection with MDR agents may increase the risk of early infection in recipients, it does not necessarily impact mortality or graft survival.^23^ As with infections caused by susceptible pathogens, the same principles apply to resistant agents, provided that effective therapeutic options are available. Donor colonization is not a contraindication for organ donation, just as the use of donors with non-bacteremic infections unrelated to the graft is acceptable, provided that these infections are controlled.^24^ In such cases, early initiation of targeted therapy for the recipient is advised, with continuation for at least seven days post-transplant. On the other hand, bacteremia and graft-related infections caused by MDR pathogens must be thoroughly assessed for recipient risk, and in many cases, donation is carefully evaluated. If the infection is well-controlled, with evident clinical improvement and available effective antimicrobial treatment, organ donation may still be feasible.^25^

Another important cause of deferral among the infectious causes observed in our study was Chagas disease. Although potential donors have been excluded due to positive serology for Chagas disease, there is no absolute contraindication for organ donation, except for the use of the intestine and heart.^26^ The risk of *Trypanosoma cruzi* transmission from donors with chronic Chagas disease in liver and kidney transplantation is lower, ranging between 10% and 20% in some case series.^27^ Organ donation can still be considered, even for seronegative recipients, provided that appropriate monitoring is in place. It is recommended to perform testing weekly during the first two months, every two weeks from months three to six, and annually thereafter or at any time following an intensification of immunosuppression.^27^ Evaluation protocols may include direct parasitemia analysis, molecular methods, pathological detection of parasites in tissues, and serology. Evidence regarding the use of prophylaxis is scarce and primarily based on case series.^27^ Additionally, prophylactic therapy may obscure early signs of infection, making rigorous monitoring the preferred approach.

Beyond reassessing the contraindications, themselves, evaluating the risk factors associated with higher non-donation rates due to infection can be an effective strategy for developing new interventions. For instance, ICU admission has been previously linked to higher donation effectiveness,^28^ and our study found a specific association with lower donor exclusion rates due to infection. This relationship may be attributed to the implementation of intensive care measures, which can reduce Healthcare-Associated Infections (HAIs). For instance, ventilator-associated pneumonia is more common in emergency intubation settings compared to intensive care units,^29^ although no association has been found with catheter-related bloodstream infections.^30^^,^^31^ The COVID-19 pandemic further increased the risk of donor exclusion due to infection, influenced not only by the virus itself but also by a rise in HAIs during this period.^32^, ^33^, ^34^

Conversely, white ethnicity was identified as a protective factor against non-donation due to infection in the overall analysis and during the non-pandemic period. Previous studies have reported higher rates of catheter-associated bloodstream infections,^35^ ventilator-associated pneumonia, catheter-associated urinary tract infections, and *Clostridioides difficile* infections among non-white individuals.^36^ It is possible that racial disparities in healthcare, differences in access to healthcare services, and differential exposure to healthcare-associated infections may have contributed to the observed association. However, the mechanisms underlying this finding could not be evaluated in the present study. Furthermore, the association was not consistent across all study periods, as white ethnicity was associated with an increased risk of non-donation due to infection during the pandemic period. Therefore, these findings should be interpreted with caution, and additional studies are needed to better understand the relationship between ethnicity and donor exclusion due to infection.

Finally, brain death due to trauma or vascular causes was also identified as a protective factor against donor exclusion due to infection. This may be associated with shorter hospitalization times and a more rapid progression to death, or even with a lower likelihood of accepting donors who died from infectious, anoxic, or neoplastic causes.

This study has limitations, including its single-center, retrospective design, the use of secondary data and potential information bias due to variability in record quality. In particular, a proportion of medical contraindications lacked detailed documentation in the secondary data sources, which may have led to incomplete classification of some cases. However, the study also has strengths, including consistency with existing literature, a large sample size, and a five-year study period, which provide robust insights into donor exclusion due to infection over time.

## Conclusion

Understanding the factors associated with non-donation due to infection is essential for developing strategies to mitigate their impact on the donation process. In our study, the primary barriers to organ donation were medical contraindications, followed by family refusal. Infectious diseases emerged as the leading cause of donor ineligibility, with higher donor exclusion rates observed during the COVID-19 pandemic. Implementing targeted strategies to address organ loss due to infections could significantly enhance donation rates and optimize organ utilization.

## Declaration of generative AI and AI-assisted technologies in the writing process

In line with Elsevier's policy on generative AI and AI-assisted technologies, the authors declare that no generative AI or AI-assisted technologies were used in the preparation of this manuscript.

## Funding

This research did not receive any specific grant from funding agencies in the public, commercial, or not-for-profit sectors.

## Declaration of competing interest

The authors declare no have conflicts of interest.

## Data Availability

The data that support the findings of this study are available from the corresponding author upon reasonable request.
